# 

**Published:** 1899-11-18

**Authors:** 


					THE HOSPITAL. '< The Standard of highest purity."? ancet. November 18th, 1899.
CADBURY'S
No. 680. Vol. XXVII. Saturday,
Nov. 18tii, 1899.
[Subscription Terms,J FRICE TWOPENCE.

				

